# Correction: Chen et al. HP3D-V2V: High-Precision 3D Object Detection Vehicle-to-Vehicle Cooperative Perception Algorithm. *Sensors* 2024, *24*, 2170

**DOI:** 10.3390/s24113320

**Published:** 2024-05-23

**Authors:** Hongmei Chen, Haifeng Wang, Zilong Liu, Dongbing Gu, Wen Ye

**Affiliations:** 1 Faculty of Electrical Engineering, Henan University of Technology, Zhengzhou 450001, China; chenhongmei@haut.edu.cn (H.C.); wanghaifeng@stu.haut.edu.cn (H.W.); 2 School of Computer Science and Electronic Engineering, University of Essex, Colchester CO4 3SQ, UK; zilong.liu@essex.ac.uk (Z.L.); dgu@essex.ac.uk (D.G.); 3 Division of Mechanics and Acoustics, National Institute of Metrology, Beijing 102200, China

## Missing Citation

In the original publication [1], Ref. [31] was not cited. The citation has now been inserted in Related Works, Paragraph 1 and should read:

Figure 1. Diagram of the three types of collaboration strategies [31]. (a) Precoordinated vehicle feature fusion process. (b) Midphase feature fusion process of the cooperative vehicle. (c) Late feature fusion process of the cooperative vehicle.

In the original publication, Ref. [40] was not cited. The citation has now been inserted in HP3D-V2V Algorithm, Point Cloud Denoising, Paragraph 1 and should read:

Given the complexity of the environment in vehicle perception tasks, LiDAR point cloud data (PCD) usually has a large amount of nonvehicle noise, such as ground noise, wall point noise, and sensor noise [40].

The order of other references in the original manuscript has been adjusted accordingly. The newly added references appear below:

31. Li, Y.; Ren, S.; Wu, P.; Chen, S.; Feng, C.; Zhang, W. Learning distilled collaboration graph for multi-agent perception. *Adv. Neural Inf. Process. Syst.* **2021**, *34*, 29541–29552.

40. Cheng, D.; Zhao, D.; Zhang, J.; Wei, C.; Tian, D. PCA-Based Denoising Algorithm for Outdoor Lidar Point Cloud Data. *Sensors* **2021**, *21*, 3703. https://doi.org/10.3390/s21113703.

The authors state that the scientific conclusions are unaffected. This correction was approved by the Academic Editor. The original publication has also been updated.
